# A Review on Flexible Electrochemical Biosensors to Monitor Alcohol in Sweat

**DOI:** 10.3390/bios12040252

**Published:** 2022-04-16

**Authors:** Nuna G. Costa, Joana C. Antunes, Antonio J. Paleo, Ana M. Rocha

**Affiliations:** 1 Engineering School and Department of Textile Engineering, University of Minho, 4800-058 Guimaraes, Portugal; a84017@alunos.uminho.pt; 2 Center of Textile Science and Technology, University of Minho, 4800-058 Guimaraes, Portugal; ajpaleovieito@2c2t.uminho.pt (A.J.P.); amrocha@det.uminho.pt (A.M.R.); 3 Fibrenamics, Institute of Innovation on Fiber-Based Materials and Composites, University of Minho, 4800-058 Guimaraes, Portugal

**Keywords:** road accidents, alcohol, biomarkers, biosensor, target analyte, biomimetic

## Abstract

The continued focus on improving the quality of human life has encouraged the development of increasingly efficient, durable, and cost-effective products in healthcare. Over the last decade, there has been substantial development in the field of technical and interactive textiles that combine expertise in electronics, biology, chemistry, and physics. Most recently, the creation of textile biosensors capable of quantifying biometric data in biological fluids is being studied, to detect a specific disease or the physical condition of an individual. The ultimate goal is to provide access to medical diagnosis anytime and anywhere. Presently, alcohol is considered the most commonly used addictive substance worldwide, being one of the main causes of death in road accidents. Thus, it is important to think of solutions capable of minimizing this public health problem. Alcohol biosensors constitute an excellent tool to aid at improving road safety. Hence, this review explores concepts about alcohol biomarkers, the composition of human sweat and the correlation between alcohol and blood. Different components and requirements of a biosensor are reviewed, along with the electrochemical techniques to evaluate its performance, in addition to construction techniques of textile-based biosensors. Special attention is given to the determination of biomarkers that must be low cost and fast, so the use of biomimetic materials to recognize and detect the target analyte is turning into an attractive option to improve electrochemical behavior.

## 1. Introduction

Road accidents are one of the main causes of death worldwide, killing around 1.3 million of people per year, particularly children and young adults [1]. The World Health Organization (WHO) predicts that by 2030 road accidents will cause 13 million deaths and 500 million injuries during the next decade, namely in low- and middle- income countries [1,2]. Furthermore, more than 20 million people per year worldwide suffer minor or serious injuries that result in temporary or permanent disability [2].

The causes of road accidents/crimes are numerous. However, driving under the influence of alcohol stands out, as the risk of suffering a road accident increases, following potential injuries with varying severity, and even fatalities. Drunk Driving is a true epidemic. Presently, alcohol is the most commonly used addictive substance worldwide, being one of the main causes of death in road accidents. Excessive alcohol consumption causes 3 million deaths/year worldwide (5.3% of all deaths), especially in the 20–39 age group (13.5% of all deaths), greatly contributing to the occurrence of accidents road fatalities [3]. Accurate and rapid measurement of ethanol is very important in clinical diagnosis and forensic analysis in order to analyze human body fluids, such as blood, urine, saliva, exhaled air, sweat, among others. A variety of methods and strategies have been reported for the determination of this analyte including liquid/gas chromatography (GC), high-performance liquid chromatography (HPLC), electrophoresis, mass spectroscopy (MS) and capillary electrophoresis (CE) [4,5,6]. However, they are inadequate processes for on-site diagnosis, not allowing real-time feedback from the person. Thus, there has been considerable interest in the development of point-of-care technologies that can analyze real biological samples and offers the possibility of rapid diagnostic results in non-laboratory settings.

The most common approach for real-time determination of alcohol intoxication currently applied is the use of breath-analyzers to indirectly estimate BAC through measurement of breath alcohol concentration (BrAC). Although this method can be applied, the resulting measurements suffer from inaccuracies and can be masked due to interferences from environmental factors such as temperature, humidity, contamination from compounds present in the mouth as well as environmental vapors [7,8,9]. Besides, the sample collection requires purposeful action by the user, which can limit the applicability of this systems. Hence, alternative strategies are needed to provide an effective, continuous, and accurate method of alcohol intake monitoring. This necessity has led to efforts on developing alcohol biosensors on wearable devices using non-invasive methods for easy monitoring and rapid assessment of an individual’s health status, being one of the great ventures in the scientific community over the past years. The utilization of electrochemical biosensors in these systems has been a major topic of such research, being the most common and widely available biosensors in the market as they shown to allow clinic analysis with near-real time monitoring capability along with highly sensitive and selective detection capabilities, presenting more successful results in comparison with other types of biosensors (optical, thermal, piezoelectric). Furthermore, their high versatility, cost-effective, simplicity of production, low detection time, simplicity of operation and ease of adaptation to wearable formats make this type of biosensors the best option to adopt in point-of-care (POC) diagnosis [10,11,12]. Wearable sensing devices have been produced to detect target analytes in sampled biofluids such as sweat, which have demonstrated a huge potential to provide a direct measure of concurrent analyte levels in blood, since it has demonstrated a high correlation between concentrations in the two fluids [13,14]. Thus, the monitoring of alcohol concentrations in sweat could be achieved without necessitating invasive blood sampling toward real-time measure of alcohol intoxication. In the literature, there are research projects related to biosensors capable of detecting ethanol in sweat, which will be presented throughout this review. The wearable/flexible platforms presented in this review represent the current state-of-the-art in wearable electrochemical alcohol biosensors. These devices have been designed to meet the needs of a variety of applications ranging from law enforcement and addiction studies to alcohol healthcare monitoring and safety.

Alcohol can be electrochemical measured in biofluids through bioreceptors (enzymes, antibodies, MIPs) [15] capable of detecting alcohol biomarkers. It can be monitored by leveraging direct ethanol oxidation or indirectly by measuring a metabolite of alcohol, such as ethyl glucuronide (EtG), ethyl sulfate (EtS), FAEEs (fatty acid ethyl esters), CDT (carbohydrate-deficient transferrin) and β-HEX (β-hexosaminidase) [16,17].

Overall, this is an unmatured market niche, with different aspects to improve and study, so this review discusses recent studies and new devices targeting on-body electrochemical alcohol monitoring with emphasis on major advances in biosensing. This review also reinforces the necessity of the scientific community take a chance on new methodologies and try, somehow create an innovative and functional product that attracts the interest of the defined target audience. Finally, our perspective on the remaining challenges and future directions of wearable/flexible electrochemical alcohol biosensors is presented.

## 2. Alcohol Biomarkers

The detection and quantification of alcohol are of great importance for research as a clinical practice, forensic scope, and use of alcohol in inappropriate environment. Ethanol absorption usually occurs by simple diffusion through the mucosa of stomach (20%) and the small intestine (80%) [18]. Ethanol is the type of alcohol found in alcoholic beverages. Around 2% to 10% of all ethanol consumed is eliminated in urine, sweat, saliva and air exhaled. About 90% is oxidized in the liver by three different pathways: Alcohol Dehydrogenase (ADH), System Microsomal Oxidation of Ethanol (SOME) and catalase signaling pathways [18]. The first methods for detecting and quantifying the ethanol level in blood were developed in 1906 by Maurice Nicloux and between 1920–1930 by Widmark, in which volatile substances, such as ethanol, were removed from the blood by diffusion in especially glass flasks followed by oxidation with chromic acid and volumetric analysis with iodometric tritation. Widmark’s method was applied to testing drivers and people who committed crimes and was used in the whole world for many following years [19]. With the development of new technologies in the extraction of analytes, the use of a variety of biological matrices has become even more accessible and possible. In addition to blood and urine, the most used ones are sweat, exhaled air, hair, saliva, and vitreous humor.

A biomarker can be considered any substance or component whose concentration found in a certain biological fluid can be used as an indicator of a certain disease or physical condition. Alcohol biomarkers have important applications in medicine and public safety. They not only provide an objective parameter of alcohol consumption to help diagnose alcohol abuse but can also be used to track the progress of diseases related to this health problem or any genetic predisposition toward alcohol abuse. The main alcohol biomarkers are:

- **Ethanol**: strongest and most studied validating biomarker. Ethanol has a short half-time in the organism, limiting its use to recent consumption. This biomarker can be detected in all biological matrices already mentioned [20].
- **EtG (ethylglucuronide)**: direct metabolite of ethanol. Although it comprises only about 0.1% of the total ethanol disposal, EtG has a wide window detection, being possible to detect this substance in blood and urine up to 36 h and 5 days after excessive consumption of alcohol, respectively. Incidental exposure to ethanol-containing products and even yeast and sugar may result in false-positives [21], while false-negatives can occur with certain urinary tract infections [22]. This biomarker has shown good results for detection through other biological matrices such as sweat and hair [23]. EtG measurements for alcohol abuse in hair have a relatively high sensitivity and specificity, of 70–90 and 80–95%, respectively [23,24].
- **EtS (ethylsulfate)**: direct metabolite of ethanol, which has similar potential to EtG as a biomarker of relapse. Both the compounds are ethanol-specific metabolic products and can be used together to detect recent alcohol use with improved sensitivity [21,25].
- **FAEEs (fatty acid ethyl esters)**: represent ester conjugates between fatty acyl chains (such as oleic acid, steric acid, and palmitic acid) and ethanol. This biomarker is detected from sebum produced through the sebaceous glands [16,17]. These glands are mostly located in the hair and face. Nonetheless, can be in all areas of the body except the palms the hands and feet. These alcohol metabolites have been reported to be present in the blood for up to nearly 100 h in heavy drinkers. The biological matrices most used for the detection of this biomarker are blood and hair [26].
- **CDT (carbohydrate-deficient transferrin)**: strong biomarker for chronic ethanol ingestion. CDT refers to the minor varieties of transferrin with lower degrees of glycosylation, including asialo-, monosialo- and disialotransferrin, which contain zero, one and two sialic residues, respectively [27]. Many versions of this glycoprotein are found in healthy people. However, studies show that consumption of alcohol increases the concentrations of this substance. The sensitivities and specificities of CDT are approximately 60–70 and 80–95%, respectively [28]. CDT levels may be influenced by other conditions unrelated to alcohol use, such as anorexia nervosa [29] and pregnancy [30]. Additionally, the measurement of CDT has been shown to be imprecise and difficult. The biological matrices most used are blood and urine [18].
- **β-HEX (β-hexosaminidase)**: is a lysosomal hydrolase that is involved in the metabolism of carbohydrates and gangliosides in the liver. After heavy alcohol consumption, lysosomes are damaged and release the enzyme into the blood stream [31]. The half-life of β-HEX in serum is approximately 6.5 days [32]. The sensitivity of serum and urinary β-HEX activity has been reported to be 69–94 and 81–85%, respectively, while the specificity of serum and urinary β-HEX activity is 91–98 and 84–96% [32]. However, elevated serum β-HEX occurs in patients with hypertension, diabetes, cirrhosis, myocardial infarction, in pregnancy and after oral contraceptive [33].

In short, the most specific biomarkers for detecting alcohol exposure are ethanol and EtG. Ethanol testing provides the most accurate determination of an individual’s alcohol level. However, this analyte is not reliably detected in body fluids beyond the first 6–12 h. Thus, there has been an increasing interest in the study of EtG as an alcohol biomarker since it is a direct metabolite of ethanol capable of detecting alcohol for a few days. EtS has similar potential to EtG as a biomarker of relapse, but studies have found higher maximum concentrations of EtG than EtS and have concluded that EtS assay is more cumbersome and provides little advantage over EtG [34]. FAEEs are elevated for up to 99 h in heavy alcohol drinkers, however it cannot be detected in the palms of the hands and feet. CDT is an indirect metabolite of ethanol and a long-term biomarker of heavy alcohol consumption. However, it cannot be tested in individuals suspected of having glycosylation disorders, anorexia nervosa or pregnant women. False-positive results for β-HEX are possible in certain circumstances, so its testing is not recommended. Alcohol biomarkers already provide substantial vital information that can be guided towards the prevention and treatment of alcohol disorders. Nonetheless, more research is needed so that biomarkers with higher sensitivity can be discovered and, in turn, limitations of currently used biomarkers for alcohol consumption can be surpassed.

## 3. Sweat as a Biological Fluid of Detection

### 3.1. Sweat Composition

Recently, there has been considerable interest in the diagnosis of sweat, that is, in the use of sweat as a non-invasive alternative to blood tests to provide data on physiology, health and human performance. Although blood remains the preferred standard in clinical, analyses of other common body fluids, such as sweat, are gaining relevance. The compositions of sweat and blood are osmotically related. Hence, similarly as with blood, the content of certain metabolites in sweat can directly reflect a disease or a physical condition. Most publications on sweat diagnosis are primarily focused on the development of skin-interfaced platforms capable of capturing and performing quantitative measurements of sweat chemistry, such as biosensors capable of detecting biomarkers like lactate [35], glucose [36], cortisol [37], adrenaline, dopamine, phenolic compounds [38,39], and electrolytes [40,41,42] in order to provide new tools for healthcare monitoring. Thus, it is essential to identify the known and unknown factors regarding the composition of sweat to generate hypotheses and guide future research in diagnosis.

**Figure 1:**
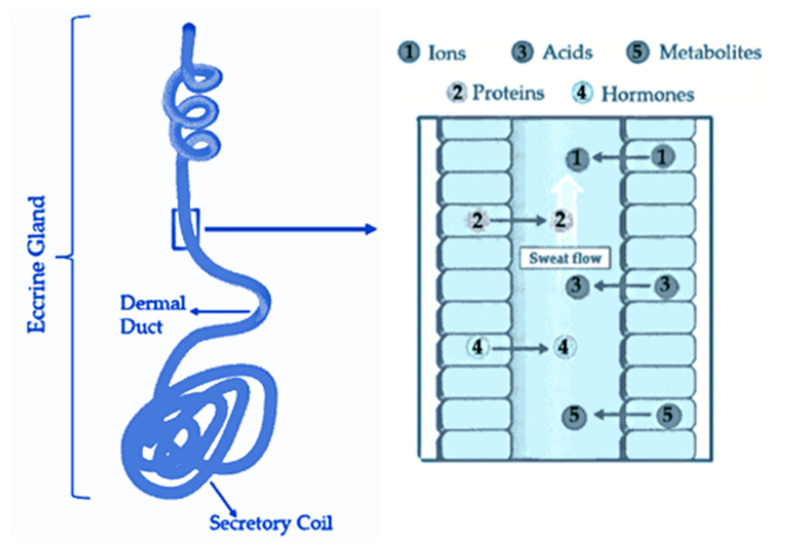
Eccrine sweat gland structure and biomarker partitioning [43].

Sweat is composed of 99% aqueous solution and a mixture of many chemicals in varying concentrations, including micronutrients (e.g., K^+^, Ca^2+^, Mg^2+^, Fe^2+^ and vitamins); metabolites (e.g., lactate, ammonia, urea, bicarbonate, amino acids, ethanol) as well as proteins and hormones such as cortisol. These compounds are mostly released through the eccrine glands. These glands are composed of a secretory spiral where sweat is generated and dermal duct, which is responsible for transporting sweat through the from the epidermis to the skin surface [43], as it illustrated in Figure 1. During this process, the referred chemicals are incorporated into sweat by passive diffusion and transdermal migration.

**Figure 2:**
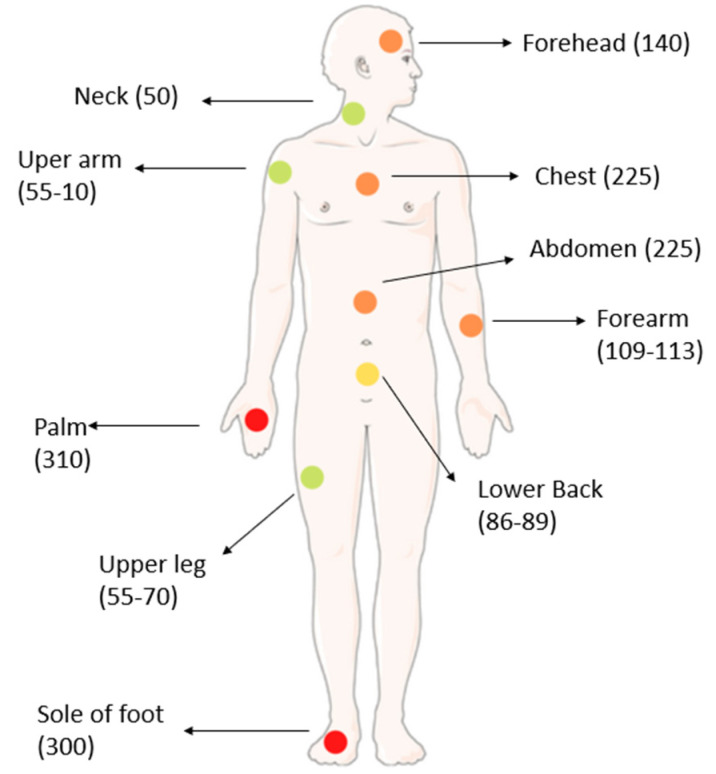
Average sweat gland density (glands/cm^2^) on various regions of the body [44].

Humans have between 2 and 5 million eccrine sweat glands throughout the body, which are more densely aggregated in certain regions, as shown in Figure 2 [44]. In general, it is usually easier detect some substance in sweat in areas of the skin with higher amounts of eccrine glands like palms and sole of foot, as they allow the analysis of larger volumes.

The correlations of chemical molecular levels in blood and sweat have been re-ported, such as glucose [45], lactate [46], ethanol [13], ammonia and urea [47]. These studies concluded that, to some extent, blood analysis can be replaced by sweat analysis.

**Figure 3:**
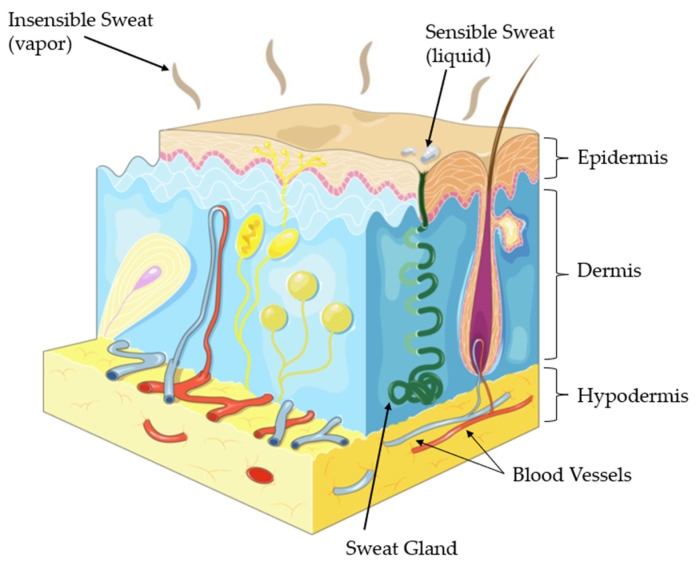
Human skin structure and two types of sweat loss in the skin surface: sensible sweat and insensible sweat [50].

However, the final composition of sweat is influenced by several factors such as extracellular concentrations solute, residual sweat, sweat flow rate, sweat gland metabolism in each area of skin, skin contamination and sample evaporation. Thus, despite all the non-invasive advantages, the diagnosis of human sweat on the skin surface of an individual may have some inconveniences such as low sample volumes, contamination of the skin and the possibility of evaporation. These challenges must be studied considering some factors such as:

- **Sweat Released Rate:** According to a study, the average sweat rate during physical activity is approximately 0.5 µL/min/cm^2^ with a range of 0.17 to 1.21 µL/min/cm^2^ [48]. When considering all areas of the skin, the resting sweat rate should be less than the sweat rate during exercise by 40% [48]. Thus, we can say that, on average, about 1.2 µL/min/cm^2^ corresponds to the amount released by an individual at rest. However, it’s important to note that the palm region, which is the study area, has a skin with higher density of sweat glands, adding ease of collection [49]. Furthermore, in addition to liquid phase sweat detection (sensible sweat), there are some devices capable of measuring volatile organic compounds (VOCs) released through skin (insensible sweat), that have shown high correlation with blood alcohol levels [24,25]. The two types of human sweat under normal conditions are illustrated in Figure 3.
- **Contamination:** Chemicals absorbed by the skin through different cosmetics can be released through sweat and interfere with detection capability. Thus, sweat should be quickly absorbed by the detection platform to avoid contamination from the skin. A way to prevent different components from interfering with the intended analyte reading is, for example, the use of a semipermeable membrane, responsible for allowing only certain substances to pass through it by diffusion [43].
- **Sample Evaporation:** it is necessary to have a fast detection in order to obtain reliable results, as evaporation acts quickly on small volumes of exposed sweat, which may change the concentration of its constituents [43].

### 3.2. Alcohol in Sweat: Correlation between Sweat and Blood

As mentioned previously, blood and urine are the most prevalent and used conventional biological matrices for carrying out alcohol consumption analyses. The use of these matrices requires the use of complex techniques such as gas chromatography [6]. Although this method produces extremely accurate BAC measurements, it uses invasive data collection methods, and it cannot be determined on site. Later, the measurement of alcohol concentration from exhaled air was developed, as it demonstrated a good correlation to blood alcohol levels.

As an alternative to blood and breath tests, sweat analysis has the advantage of containing compounds capable of serving as biomarkers without the need for standard invasive testing methods. In addition, through the analysis of the alcohol content in sweat, the results cannot be altered in the same way as with the breathalyzer method, so there is a lower risk of tampering by interference with foods/drugs taken orally. Circulating in the blood stream, ethanol diffuses into surrounding tissue, including the skin, being the amount of direct excretion of unchanged alcohol in sweat approximately 1% [51]. This allows the concentration of ethanol in sweat to be used as an indicator of the presence of ethanol in blood. However, as alcohol does not diffuse through the skin instantly, there may be a slight delay in the corresponding values of its concentration in blood and sweat.

With the aim of making meaningful interferences about a person’s state of heath it is extremely important to correlate the concentrations of the target analyte (biomarker) in sweat and blood.In doing so, the literature advocates for the study and development of sweat alcohol detection devices to compare the ethanol concentrations obtained in sweat and blood. One of the first studies was carried out by Buono [13], where it was possible to demonstrate that the ethanol concentration in sweat is highly correlated with blood ethanol concentration. In this study, sweat and blood samples were collected from ten volunteers 1, 2 and 3 h after the ingestion of approximately 13 mmol of ethanol diluted in a 15% fruit juice solution over a period of 30 min. These variables revealed a Pearson correlation of 0.98 indicating a linear variation [52] Furthermore, the slope obtained was 0.81, meaning that the ethanol concentration in blood corresponded to 81% of the ethanol concentration of ethanol in sweat. During this period, Kamei et.al [14] proposed a novel instrumentation capable of estimating and comparing the ethanol concentration in sweat and blood. This proposal consisted in a sampling probe attached directly on the surface of the skin, where it is possible to measure the rate of sweat released, as well as the ethanol concentration in sweat. Three volunteers participated in this study, where each drank 700 mL of beer with 5% ethanol in a period of 2 to 5 min. During this process, the concentration of ethanol in blood was simultaneously measured through of an authorized clinical method. From the obtained results it was possible to conclude that the concentrations of ethanol in blood and sweat reached the maximum peak at almost the same time, demonstrating a very similar profile after ingestion of alcohol by humans. Other scientific studies, carried out by Nyman and Palmlov have reported that the concentration of ethanol in sweat is about 15% higher than the concentration of ethanol in blood [53] The common conclusion in all these studies concerns the high correlation between these concentrations (Pearson correlation indexes very close to 1). Moreover, they also conclude that the concentration of ethanol in sweat is higher than found in blood by approximately 15% [13,14,53]

It is important to highlight that for a good evaluation there are some factors that can influence the action of alcohol such as body weight, dose of ethanol ingested and metabolic rate. Studies have shown that body weight has little effect on the time interval between peak BAC and TAC (transdermal alcohol concentration) and for a specific dose, the peak alcohol increases as body weight decreases. Regarding the rate of metabolism, the lower the metabolic capacity, the higher the concentration of ethanol present in the body, resulting in a longer peak delay [51].

### 3.3. Flexible Biosensors to Detect Alcohol in Sweat

A biosensor is a device capable of providing biometric data, including the concentration of certain chemical substances present in biological fluids, such as alcohol in sweat.

The interest in wearable biosensors has increased recently, since biosensor’s flexibility has begun to attract considerable attention to the scientific community. Transdermal biosensors have been designed in tattoos/skin patches, shirts, and other flexible substrates such as woven fabrics or polymer-based manufactured fibrous structures. Before the description and presentation of some projects about the development of flexible electrochemical biosensors for alcohol monitoring, Table 1 shows an overview of the most representative electrochemical sensors to detect alcohol intoxication through sweat. Some of them are available today.

**Figure 4:**
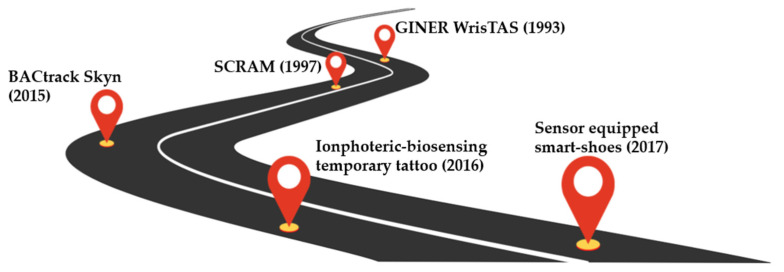
Roadmap of Flexible Electrochemical Biosensors for Alcohol Monitoring.

In the past decade, the field of wearable and flexible biosensors has seen substantial development, and most applications are health-related through the transduction of physical parameters. The development of biosensors has brought a new era of advances in science. Of the electrochemical devices under evaluation, only the SCRAM™ unit is commercially available at present, but the technology underlying the devices mentioned above are already commercially viable. In order for that to be perceptible, the development of different wearable electrochemical biosensors (flexible and non-flexible) is presented in Figure 4 as a roadmap with the evolution of these devices over time. The SCRAM™ is designed as an ankle bracelet with a sensor compartment and a digital signal processing compartment, which transmits the collected data to an in-home modem [54]. This platform has been implemented by law enforcement personnel to monitor individuals with alcohol-related offenses. The WrisTAS^TM^ corresponds to the first wrist bracelet designed for use in medical settings to control alcohol abstinence [55]. In 2015 a new generation wrist-worn bracelet for alcohol monitoring with came out, and connects via Bluethooth to an app on a user smartphone [56]. Since then, wearable sensing devices have been designed to detect alcohol detection through flexible platforms, such as temporary tattoos [59].

As previously mentioned, in recent years, some alcohol detection systems have been developed in the analysis of sweat, such as attempts to obtain non-invasive, continuous, and discrete alcohol sensors. Currently, flexible electrochemical biosensors are the most commonly used in biofluids analysis, where its principle is based on the reaction of the bioreceptor (enzymes, antibodies; etc.) with the target analyte in order to obtain an electric response (current, potential difference).

In 2016, a wearable electrochemical biosensor capable of monitoring alcohol consumption by detecting and quantifying a direct metabolite of ethanol in sweat, the EtG, was developed [23]. To this end, in this study, two coplanar sensors were developed with gold (Au) and zinc oxide (ZnO) integrated into polyimide (PI) by bonding. Up until then, it was possible to detect EtG in sweat using complex techniques such as gas chromatography associated with mass spectrophotometry, however they were deemed and inadequate processes for on-site diagnosis, not allowing real-time feedback from the person [6,9]. Thus, this project allowed to monitor alcohol consumption by detecting EtG in sweat through wearable biosensors, reacting with colour change through a LED in the presence of EtG in human sweat. Regarding to the results obtained, the ZnO sensor showed a detection capability in the concentration range of 0.001–100 μg/L up to 4 h. On the other hand, the Au sensor demonstrated an ability to detect EtG in the concentration range of 1–10,000 μg/L up to 9 h [23]. The authors concluded that the biosensor could detect ingestion of alcohol up to 11 standard drinks in the United States for a period of 4 to 9 h [23]. In 2019, the chemist Jan Halamék and his team at the University of Albany in New York aimed ai developing a new non-invasive method to assess the level of alcohol content in the blood of an individual based on the presence of ethanol in sweat. To do so, 26 volunteers of different ages, genres and eating habits participated [48]. This detection system uses a polyethylene strip composed of two enzymes, alcohol oxidase, and horseradish peroxidase in order to relate blood ethanol concentrations with sweat ethanol concentrations from a series of biochemical reactions. As soon as the strip meets the skin, the chemical reaction of the two enzymes with the ethanol in sweat produces a color change, resulting in a blue-green tone, increasing its intensity with the increasing concentration of ethanol in the analysis sample. The present study allowed the quantification of ethanol in the human sweat of 26 volunteers as how to prove that as the individual ingests alcoholic drinks, the concentration of ethanol in sweat increases linearly with the concentration of blood alcohol levels [48]. Some research groups have integrated the pilocarpine iontophoresis process into the biosensors of to stimulate sweating and monitor alcohol concentration in induced sweat. This is a transdermal administration of pilocarpine followed by amperometry detection of ethanol. In the study developed by Jan Halámek the same process was only used to obtain the amount of sweat needed for the initial proof of concept corresponding to 8μL, where concentrations were added millimolar ethanol (Mm) of 0; 10.85 and 17.35 corresponding to 0% BAC; 0.05% BAC and 0.08% BAC, respectively [48]. Kim et al. developed a flexible portable biosensor consisting of a temporary tattoo, which adhered to the skin in order to accurately measure the level of blood alcohol through ethanol concentrations in human sweat [55]. This biosensor integrates a substance capable of inducing sweat through iontophoresis, pilocarpine, enabling the amperometry detection of ethanol in sweat by the enzyme AOx. Regarding the results, the biosensor exhibited a highly selective and sensitive response to ethanol. The bodily effects in humans have shown significant differences in the current response before and after alcohol consumption, reflecting increased levels of ethanol. The device was considered more effective in relation to the Breathalyzer method, since it avoids possible inaccuracies caused by changes in temperature, humidity, environmental factors such as alcoholic vapors or such as foods/drugs taken orally, resulting in a lower risk of tampering [59].

A summary of the flexible biosensors discussed above is given in Table 2.

## 4. Biosensors

### Components of a Biosensor

The construction of a biosensor requires multidisciplinary research in engineering, physics, chemistry, and biology. The choice of materials and methods generally depends on the target biomarker, especially in its molecular properties and concentration range.

A biosensor is composed by the bioreceptor [e.g., enzymes, antibodies, aptamers, molecular imprinted polymers (MIPs), etc.], the transducer and the processing system [58,62].

**Figure 5:**
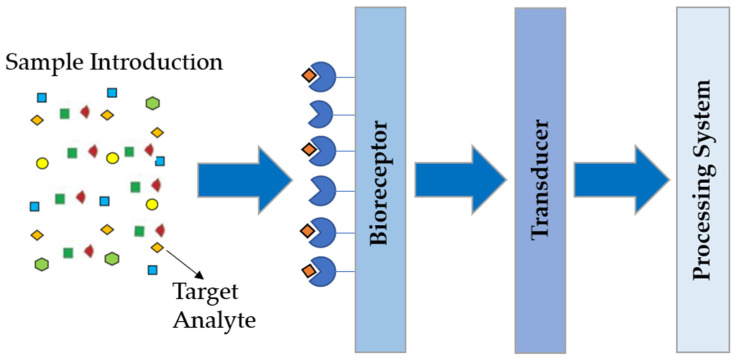
Schematic of the main components and mechanism of a biosensor [63].

The bioreceptor is responsible for interacting with the target analyte (biomarker), in order to produce an effect capable of being detected and measured by the transducer, which transforms it into a signal proportional to the presence of the target analyte in a sample. This signal can be viewed, amplified, and stored through the processing system [63]. The scheme relating to components of a biosensor is presented in Figure 5

Biosensors can be classified according to the transducer used:

- **Optical biosensors**: analytical devices composed of a biorecognition element integrated into an optical transducer system and its main aim is to produce a signal that is proportional to the concentration of the substance measured (target analyte) [64,65].
- **Thermal biosensors**: use heat generated by exothermic enzyme catalytic reactions to measure the concentration of the analyte. Temperature changes are usually determined by high-sensitivity thermistors. These biosensors are not easy to handle [64,66].
- **Piezoelectric biosensors**: are based on the piezoelectric property which the anisotropic crystals (quartz, for example) possess. When an alternated voltage is applied to this biosensor, the crystal oscillates with a certain frequency, associated with the mass and elastic constants of the crystal [67].
- **Electrochemical biosensors**: have the aim of generating an electrical signal that is related to the concentration of the target analyte in the sample. The chemical reactions that occur between immobilized bioreceptor and the target analyte produce or consume ions or electrons affecting electrical behavior, such as current or electrical potential [64,68].

**Figure 6:**
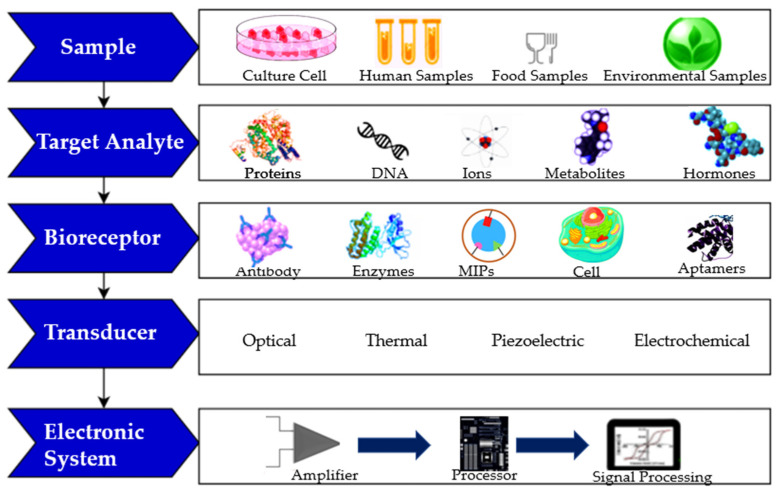
Possibilities of different components of a biosensor [49].

The different possibilities of samples to be analyzed; target analyte; bioreceptor and transducer type are represented in Figure 6.

Electrochemical biosensors, compared to the different types of biosensors described above, are the most common ones as they shown to be the most successful in biofluid analysis, given their high versatility, low cost, simplicity of production, high sensitivity and selectivity, low detection time, simplicity of operation and ease of adaptation to miniaturization, portability and wearable formats. Electrochemical biosensors are classified according to how the analyte is measured, that is, with the electrochemical technique used to measure the resulting current [12,69,70].

## 5. Electrochemical Techniques

Electrochemical techniques are used in order to analyze the loss (oxidation) or gain of electrons (reduction) that given material undergoes during an electrical stimulus. These oxidation-reduction (redox) reactions provide information such as concentration, kinetics, reaction mechanisms and other behaviors of a specie in solution [68]. That said, from electrochemical measurement it is possible to obtain a wide range of electrical properties such as potential, current, charge and time, allowing to evaluate the performance of a biosensor.

As stated before, electrochemical biosensors can be classified according to the characteristics of the signal obtained by transduction. Thus, according to the type of signal, which may be potential difference, current intensity or impedance/conductance changes, biosensors can be classified as potentiometric, amparometric and impedimetric, respectively [69].

- Potentiometric: potentiometric biosensors measure the potential difference of the working electrode and the reference electrode. This potential difference is formed when, for example, an antigen-antibody interaction occurs, and it is measured under practically zero current conditions [64,70].
- Amparometric: amparometric biosensors measure the current produced due to electrochemical oxidation or reduction of electroactive species at the working electrode when a constant (in case of chronoamperometry measurements) or a variation (in case of voltammetry measurements) of potential is applied to the working electrode with respect to the reference electrode. The measured current is the rate of transferred electrons as a function of time, being proportional to the concentration of the target analyte [71].
- Impedimetric: impedimetric biosensors measure the electrical impedance produced at the electrode interface when a small sinusoidal perturbation signal is applied. It involves the application of low amplitude AC voltage at the sensor electrode and then the current response is measured as a function of frequency using an impedance analyzer [64,72].

**Figure 7:**
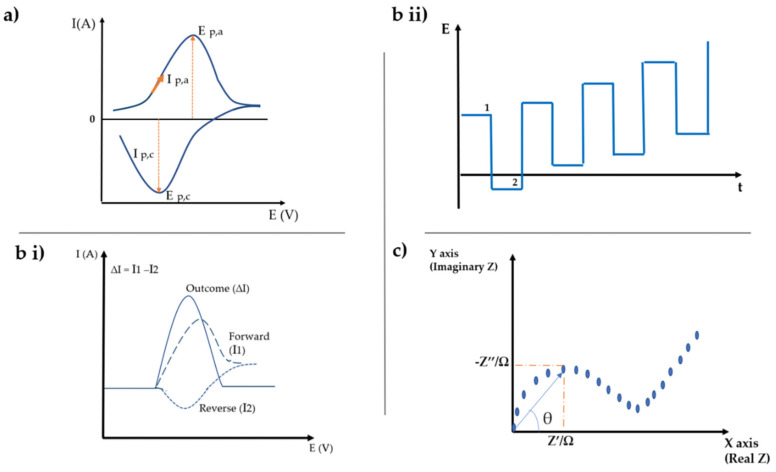
(**a**) —Typical voltammogram for a reversible system, where Ipa is the Anodic peak current and Ipc is the Cathodic peak current [74]. (**bi**)—Scheme of application of potentials: sum of a staircase and a square wave [74]. (**bii**)—Schematic square wave voltammogram of a redox reversible process [74]. (**c**)—Nyquist plot that illustrates both real (Z′) and imaginary (Z″) components of impedance [68].

Among the most used electrochemical techniques, amparometric techniques such as chronoamperometry and voltammetry and electrochemical impedance spectroscopy (EIS) as an impedimetric technique stand out. The typical graphs of these techniques are illustrated in Figure 7. Typically, these techniques require connecting three electrodes (reference electrode (RE), auxiliary electrode (AE) and working electrode (WE)) in order to determine the potential of the working electrode and measure the resulting current [68,73]. Techniques such as voltammetry and EIS have been utilized for the detection of drugs and hormones.

### 5.1. Voltammetry

The amparometric techniques that depends on the measurement of current, depending on the applied potential, are called voltammetric techniques. The selection of the voltammetric technique to be used depends on the type of quantitative/qualitative information to be obtained.

#### Cyclic Voltammetry (CV)

Cyclic Voltammetry is the most common electrochemical technique because of its simplicity and speed.

From Figure 7a it is possible to observe a variation of the current along an oxidation process which occurs from the initial potential to an inversion potential. In this region, the potential is subject to a positive sweep, resulting in an anodic current (I_p,a_), with oxidation of the species studied and, consequently, an increase in current. On the other hand, after the inversion potential is reached, the sweep potential becomes negative, resulting in a cathodic current (I_p,c_) and reduction occurs [74,75].

Although it is not frequently used in quantitative analysis, it allows us to obtain qualitative information on any electroactive species, such as the reversibility of systems, as well as the occurrence of adsorption of products on the working electrode, with an increase in the intensity of the current with the concentration of the analyte to be analyzed [76]. The greater the reversibility of a system, the more sensitive the measurement will be [76].

In a reversible reaction, the total amount of oxidized species at the anodic peak will be identical to the amount of species reduced in the cathodic peak. Furthermore, the cathodic and anodic peak currents should be proportional to the concentration of the substance being reduced and oxidized, increasing and decreasing, linearly with the square root of the sweep speed.

The general shape of a voltammogram depends on the reversibility of the redox pair on the electrode surface. Reversible systems also allow to obtain quantitative data through the Randles Sevcik equation from the intensity of the peak current (*i_p_*), as shown in Equation (1) [76].

(1) $$i_{p}=2.69\times 10^{8}n^{3/2}AD^{1/2}V^{1/2}C$$

where,

*n* = *number of electrons involver*;

*A* = *electrode area* (m^2^);

*D* = *difussion coefficient* (m^2^/s);

*V* = *sweep speed* (m/s);

*C* = *concentration* (mol/L);

### 5.2. Square Wave Voltammetry (SWV)

When applied to reversible systems, square wave voltammetry, enables us to obtain more intense signals, allowing an increase in sensitivity compared to other voltammetric methods. It is closely related to the analysis of molecules biological techniques, since it is one of the fastest and most sensitive techniques, capable of obtaining limits of detection very close to those of chromatographic techniques, which are extremely accurate. In general, it allows to obtain a good quantitative analysis through the obtained calibration curves, being possible to obtain very low detection limits, in the order of 10^−7^–10^−8^ mol/L [77].

In this technique, pulses of potential are applied, the current being measured twice in each cycle, at the end of the forward pulse and at the end of the inverse pulse, obtaining a signal in the form of a ladder, as it shown in Figure 7(bii)).

The resulting voltammogram demonstrates the resulting current obtained, that is, the difference between the direct and inverse currents, this being the one used for analytical studies, as it shown in Figure 7(bi)).

### 5.3. Electochemical Impedance Spectrocopy (EIS)

The EIS allows obtaining information about the different time constants associated with the electrochemical processes that occur at the electrode interface. In this technique, a small sinusoidal perturbation is applied to the working electrode and its response when it is in steady state is recorded. Normally, the disturbance is applied to the potential and the resulting current is measured. In general, the EIS analyzes the variations in the electrical transfer charges of the redox system that occur at the electrode of work through each modification step. This technique is used to characterize the resistance, corresponding to the ability of a circuit to resist the flow of current [72].

The presence of the bioreceptor on the electrode surface occurs when there is an increase in the resistance of electron transfer. Impedance is the proportionality factor between the Potential (E) and Current *(I*) over time, being measured in Ohm, as represented in Equation (2).

(2) $$Z=\frac{E\left(t\right)}{I\left(t\right)}\;\Omega$$

The impedance can be evaluated using the Nyquist plot, as it shown in Figure 7c. This presents a semicircle in a region of higher frequencies, which means a controlled behavior of the charge transfer. This semicircle is followed by a straight line at lower frequencies, which indicates a diffusion-controlled behavior. An increase in the size of semicircle corresponds to an increase in charge transfer resistance [76].

In addition to the four techniques mentioned above, Table 3 summarizes all the possible techniques to use for the detection of a specific target analyte in electrochemical biosensors, and their respective advantages and disadvantages.

## 6. Types of a Bioreceptor

### 6.1. Enzymes

Enzymes are proteins capable of catalyzing a biochemical reaction and its use gives rise to enzymatic electrochemical biosensors. They selectively react with a target analyte, accelerating the reaction and providing an alternative reaction pathway with a lower activation energy [78]. Enzymes are responsible for recognizing the target analyte, capturing it, and catalytically converting it into a product measurable, which is usually monitored using amperometric transduction methods [79]. The use of enzymes as a bioreceptor has the advantage of promoting biosensor selectivity. However, as they depend on the catalytic activity of the medium, variations in pH, even if they are minimal, may condition or limit the enzymes catalytic activity. In relation to biosensors for the detection of alcohol, it is verified, so far, the use of alcohol oxidase enzymes (*AOx*), horseradish peroxidase (*HPR*) and Alcohol Dehydrogenase (*ADH*) as a bioreceptor.

The reaction presented in Equation (3) demonstrates the ability of the AOx enzyme to catalyze the oxidation of *Ethanol* to generate hydrogen peroxide, capable being detected electrochemically through of the transducer [7,80].

(3) $$Ethanol+O_{2}\overset{AOx}{\to }H_{2}O_{2}+Acetaldehyde$$

The enzyme *ADH* catalyzes the reversible oxidation of *Ethanol* to form *Acetaldehyde* with coenzyme nicotinamide adenine dinucleotide (*NAD*), as shown in Equation (4) [7,80].

(4) $$Ethanol+NAD+\overset{ADH}{\Leftrightarrow }NADH+Acetaldehyde+H^{+}$$

HRP is normally used in combination with AOx enzyme to promote the electron transfer. It is an enzyme capable of oxidizing organic substrates, being hydrogen peroxide being the electron-accepting molecule, as shown in Equation (5) [4].

(5) $$H_{2}O_{2}+p-fluoraniline\;\overset{HRP}{\to }F^{-}+H_{2}O+aniline\;derived\;polymers$$

### 6.2. Antibodies

Together with DNA, antibodies, due to their three-dimensional structure, are considered the more selective biological agents, creating a unique recognition pattern, presenting high specificity and precision for the target analyte. Biosensors that use antibodies as an element of biological recognition are called immunosensors. Thus, an immunosensor is a type of biosensor which detects the specific target analyte called antigen by the formation of a stable immunocomplex between the antigen and the antibody as a capture agent, generating a measurable signal obtained by the transducer [81]. The disadvantage of using these elements in biosensors is the fact that it has low stability and high cost. In the literature, so far, one finds references to biosensors for detecting alcohol through the use of the EtG antibody as a bioreceptor.

### 6.3. Molecularly Imprinted Polymers

Usually, the integration of natural bioreceptors into biosensors causes low stability and durability, since they end up having little resistance to adverse environments such as high temperatures, high pressure and pH variations. In addition to being unstable outside it natural environment, small amounts may exist or even not exist for a given target analyte. Thus, an alternative technique called Molecular Printing has recently emerged, allowing the creation of these structures in an artificial way. It consists of a technology capable of producing polymers provided with specific recognition locations, molded from a template molecule, which may be the analyte itself or a similar structure [82]. Comparing with systems that use natural bioreceptors such as enzymes and antibodies, this technology has advantages such as low cost, robustness, high stability, long-term durability and selectivity [83]. Regarding to the development of alcohol biosensors from molecular imprint polymers (MIPs) as a bioreceptor the literature is limited.

**Figure 8:**
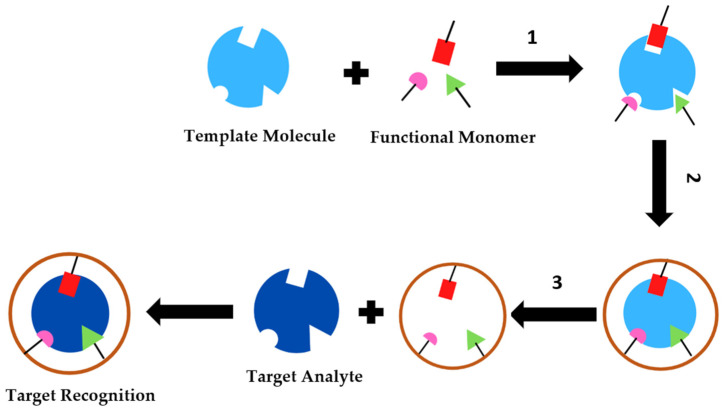
Schematic representation of the molecular imprinting process. 1—complexation; 2—polymerization; 3—extraction [85].

In general, the synthesis process of a MIP includes three main steps: complexation (1); polymerization (2) and extraction (3), as shown in Figure 8. The first step allows the functional monomers to interact with the template molecule, forming a complex (template/monomer) through covalent or non-covalent bonds. The second step concerns the polymerization of the monomers present in the complex through a cross linker, forming a rigid polymer. This polymerization reaction is started after the addition of a radical initiator and the mixing of polymerization usually has a solvent that induces the formation of pores in the polymer (porogenic solvent) giving rise to a rigid polymer matrix. Finally, the third stage deals with the extraction of the template from the polymeric matrix by successive washing processes, in order to break the bonds between the template and the polymeric matrix, giving rise to the specific cavities [84]. These cavities give the MIP the ability to selectively recognize and retain the initial molecule present in any sample complex [82].

During the process of synthesizing a MIP, factors such as stoichiometric ratios of the components, temperature and time of the polymerization reaction have a significant impact on chemical and morphological properties features exhibited by the MIP, and, therefore, all these variables must be carefully studied in order to obtain maximum efficiency [83,84].

The template molecule must fulfill a set of requirements, namely it be chemically inert; remain stable at moderately elevated temperatures or upon to exposure to UV radiation; and it may not have groups that accelerate or retard polymerization (e.g., thiol) as well as polymerizable groups, since these promote the non-formation of recognition cavities. Furthermore, this component should be able to bond with the functional monomer otherwise there will be no printing [83].

The functional monomer is responsible for the interactions of the chemical bonds in the imprinted binding sites. Therefore, it is necessary to combine the functional groups of the monomer with the functional groups of the template molecule. Thus, a template that has basic functional groups (proton acceptor) interacts more easily with a monomer that contains acidic functional groups (pronto donor), and vice versa [86]. Methacrylic acid (MAA) has been used as a universal monomer due to its good appetite for hydrogen bonds and ionic bonds. However, knowing that the selection of the most suitable monomer depends on the template molecule, many studies refers to MAA and vinylpyridine (VP) as the most used in the synthesis process for basic and acid molecules, respectively [81,84,85]. This component must always be present in the mixture, in an amount greater than the molecule template in order to guarantee the formation of as many specific cavities as possible. The most common ratio is 1:4, however, the molar ratio between these two components must be well studied since much higher amounts of monomer may form non-specific cavities [81].

The cross linker is extremely important to provide mechanical stability to the polymer matrix. This component must be present in concentrations higher than the monomer, the molar ratio is generally 1/5 in order to guarantee the porosity of the polymer and generate materials with adequate mechanical stability. Quite a number of cross-linkers compatible with molecular imprinting are known, such as ethylene glycol dimethacylate (EGDMA); divinilbenzen (DVB) and trimethacrylate (TRIM) [86,87].

The initiator has the function, as its name implies, to enable the start of the reaction through the formation of free radicals. The most commonly used has been Azobisisobutyronitrile (AIBN). The beginning reaction also needs a physical stimulus such as temperature or UV radiation [83]. In general, this component is used in low amounts compared to the functional monomer, corresponding to about 1% of its mass [85].

The solvent corresponds to the medium in which all the components involved are found. In the synthesis of an MIP (template molecule, functional monomer, cross linker and initiator), the thermodynamical properties of the solvent with the other compounds develop highly porous structures and larger specific areas, being, therefore, necessary to consider the nature and volume of this component in the molecular printing process [87]. Moreover, the solvent must guarantee the morphology polymer without interfering with the monomer-template bond. In non-covalent imprinting polymerization, the solvent should be apolar, aprotic (e.g., toluene, chloroform, dichoromethane) since that are capable of stabilizing hydrogen bonds.

Oxygen gas retards free radical polymerizations, since it can cause the formation of an excess of radicals, which can negatively influence the whole process. Thus, with the aim of maximizing the reproducibility of polymerizations, the removal of dissolved oxygen must be carried out by bubbling the solution by an inert gas, such as nitrogen or argon [88].

Normally, polymerization is carried out at a temperature of 60 °C. However, recent studies have demonstrated that it is possible to obtain more selective MIPs when lower temperatures are used in the polymerization step, using initiators that are photochemically activated [89].

The longer the polymerization time, the more rigid are the produced polymers are. Although rigid polymers contain specific cavities of molecular imprinting that are better defined, giving rise to MIPs of greater specificity, this property may cause a decrease in mass transfer and, in turn, in binding kinetics [90].

## 7. Immobilization Techniques

The bioreceptor immobilization is a process by which it is deposited on the transducer, which can be achieved by covalence (a); adsorption (b); crosslinking (c); entrapment (d) among others, as illustrated in Figure 8. The main objective of the immobilization is to retain the activity of the bioreceptor, so it is crucial that the biosensor is sensitive and selective for the target analyte in order to avoid interference of other substances present [91].

Table 4 presents a brief overview on some advantages and limitations of different immobilization methods.

In addition to the techniques mentioned above, when using conductive ink it is possible the immobilization of bioreceptors through the formation of composites. This has been approached as an attractive technique due to its simplicity of execution. It is used in cases where the electrodes are produced from conductive ink. The process basically consists of adding the bioreceptor to the ink that will constitute the working electrode [96]. It has presented advantages such as ease of preparation, low detection limits, stability and selectivity. However, an inhomogeneous mixture can lead to limitations in binding kinetics [99].

## 8. Requirements of a Biosensor

A biosensor must be highly specific, independent of physical parameters such as pH and temperature and must be reusable. In order to allow for the robust and accurate quantification of analytes in the sweat, there are several attributes of the biosensors must be optimized. Such attributes/requirements include sensitivity, selectivity, detection limit, stability, response time and reproducibility [43,62,100].

- **Selectivity**: main requirement of a biosensor. It deals with the ability to recognize and detect the target analyte in the presence of other potentially interfering species. For such, biological recognition elements are immobilized on the surface of the transducer [101]. A factor that influences selectivity is biofouling, that is, accumulation of chemical species in the detection layer that gradually degrades the performance of the sensor. This effect can be reduced with the incorporation of semi-permeable membranes like cellulose acetate, for example and detect the target analyte in the presence of other potentially interfering species.
- **Sensitivity (*S*)**: it is the measure of how sharply the signal changes with variations in the concentration of the target analyte. Calibration curves can be generated with the gradual increase of the concentration of the analyte with the aim of determining the sensitivity of the sensor through the slope of the curve obtained in cases where hysteresis is insignificant, as it shown in Equation (6). In the case of drug detection systems may only require binary information to determine the presence or absence of a drug above a predefined tolerance level. In such cases, the sensor does not need to have a very high sensitivity but should be able to distinguish concentrations above or below the threshold [101]. The use of materials such as carbon nanotubes or metal nanoparticles contributes to a better transfer of electrons and thus amplify detection.
  (6) $$S=\frac{dY}{dX}\;\;\;\;$$
- **Detection Limit (*LOD*)**: indicates the lowest concentration which a sensor can detect and stems from signal-to-noise relationships. The signal noise-ratio with a value of 3.3. is generally acceptable [101]. Thus, the limit of detection (*LOD*) may be expressed by the Equation (7):
  (7) $$LOD=\frac{3.3\times s}{S}\;\;\;$$

Improving detection limits requires amplifying the target signal to suppress background noise, both of which can be achieved with the materials rights and detection schemes. Sensors must be designed to have their detection limit below the physiologically relevant concentration range of the analyte target. Low detection limits can be achieved from electrochemical deposition of conductive polymers such as polypyrene, for example. Detection limits enzymes and voltametric sensors can be reduced using nanoparticles and nanostructures in order to improve binding affinity and increase the number of sites of reaction.

- **Stability:** refers to the sensor ability to maintain the signal over time. Hydrophobic materials can improve this property and conductive polymers such as polystyrene, polypyrrole and polyaniline improve even more stability. In addition, the immobilization of the bioreceptor through techniques such as crosslinking with polymers help to improve this ability. A balance must be achieved in order to optimize stability and sensitivity.
- **Response time:** sensor response time to stabilize at a reliable value when the concentration of the analyte change. Response times are typically influenced by target analyte, bioreceptor and sample composition. A lower thickness of the bioreceptor helps to decrease the response time.
- **Reproducibility/Precision:** the degree of reproducibility required depends on the sensitivity of the sensor [101]. Screen printing techniques allow the production of high-yield sensors with high uniformity. Refers to the ability of the sensor to maintain the signal over the time. The proximity between several measurements performed on the same sample in different days/conditions is generally obtained through the coefficient of variation (*CV*) expressed by the Equation (8).
  (8) $$CV\;\%=\frac{S}{M\;}\times 100$$
- **Linearity**: it is necessary to use enough standard solutions (minimum 5) of to adequately define the relationship between concentration and response. Linearity refers to the ability to generate results linearly proportional to the concentration of the target analyte. This parameter can be obtained through the Pearson correlation index acquired through the calibration curve performed. It is known that the closer to one the absolute value of this coefficient is, the stronger the linear relationship between the two variables to analyze is [101]. The linear range corresponds to the target analyte concentration range the obtained response is linear.

## 9. Construction of Flexible Biosensors

In recent years there has been growing interest in the construction of electrochemical biosensors, as it allows to obtain and signal with minimal manipulation of the system. They are usually small, low-cost, easy-to-use devices providing quick on-site response through a small volume sample.

The integration of electrochemical biosensors in textiles has been the object of growing study due to the fact that it is a platform with several advantages such as non-invasive and highly sensitive alternatives to analysis of several physiological parameters. These have the ability to respond to a given analyte selectively through an electrochemical reaction. For this to happen, there must be changes to the sensor surface when adding the bioreceptor. The bioreceptor and the transducer constitute the working electrode and the results obtained are related with mobility of charges and/or oxidation-reduction reactions that occur on its surface.

Substrates such as polyamide (PA), polyethylene terephthalate (PET), polytetrafluorethylene (Teflon), polyester (PES), polyaniline (PANI), polyvinyl chloride (PVC), polypropylene (PP) among other, have been used for a long time in the electronics industry for the development of textile sensors [100]. This is due to your intrinsic plasticity, hydrophobicity, thermal stability, low coefficient of thermal expansion, structural resilience again repeated bending forces and excellent dielectric properties [102].

Printing techniques have been used for the construction of electrochemical biosensors, and the screen printing is the most used [63]. However, inkjet and gravure printing have also been used to produce sensors in a quick and simple way. Recently, there has been substantial growth in the development of flexible biosensors for monitoring the person’s physical condition. In addition to monitoring alcohol consumption, there are biosensors capable of detecting viruses, diseases such as diabetes, cardiac insufficiency, parkinson/adrenalide.

The characteristics of these biosensors are summarized in Table 5.

### 9.1. Screen Printed Electrodes

**Figure 9:**
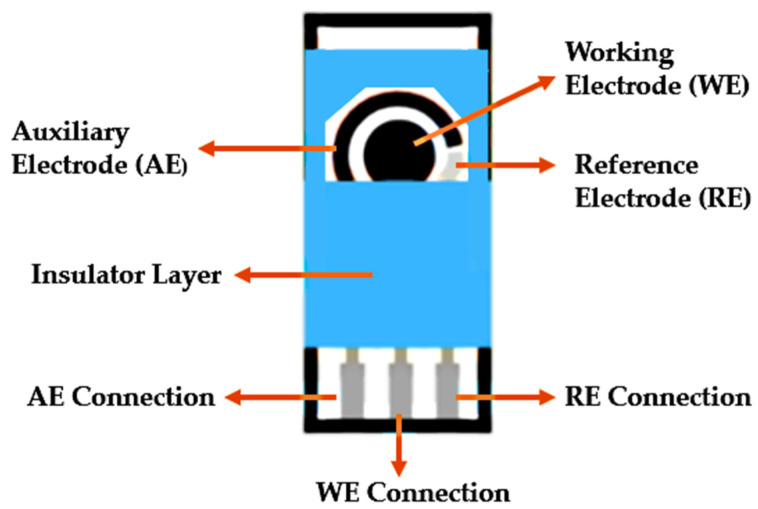
Typical design of an electrochemical biosensor produced by screen printing [109].

One of the most used techniques in the design of electrochemical textile biosensors is based on the Screen Printing, which consists in the production of Screen-Printing Electrodes through the deposition of layers of ink onto a substrate through a screen. In order to obtain the design requested in the print screen is normally produced a stencil, allowing the selected image to pass to the textile substrate [107]. This has been shown to be a quite attractive technique, as it has several advantages over the other manufacturing processes such as greater design flexibility, process automation, good reproducibility and the possibility of using a wide variety of materials [108]. In addition, its miniaturized size and the possibility of integration into portable devices make it possible to detect at the location and time of a specific target analyte. The most relevant difference between this printing technique and others is that from screen printing it is possible to print films of high thickness, which may influence the electrochemical behavior of the biosensor, improving it. For the construction of SPEs, it is usually necessary to configure three electrodes in order to form an electrochemical cell: reference electrode, working electrode and auxiliary electrode (counter electrode) as shown in Figure 9.

The reference electrode (RE) is used for the purpose of giving a fixed potential value to which other potentials can be referred to in terms of a potential difference. For a good functioning of the biosensor, it is necessary that the reference electrode has a stable potential with time and temperature and that is not altered by a small current flow. A good example is the silver electrode/silver chloride (Ag/AgCl) [110].

The working electrode (WE) is what makes it possible to control the potential and measure the intensity of the electric current. The bioreceptor must be immobilized on its surface. This material must be inert and stable in working potentials such as carbon, gold, zinc oxide, platinum, gold [110].

The auxiliary electrode (AE) has the function of closing the circuit in the cell. This must be an inert conductor like graphite, for example. It must have a surface superior to the working electrode in order to allow a electron transfer with minimal overvoltage [110].

#### SPEs Process of Formation: Substrates, Inks and Additives

Currently it is possible to acquire different substrates, conductive inks and additives for the production of SPEs focused on research and innovation. According to the literature, the use of nanomaterials for the construction of SPEs has shown excellent results. Depending on the intended use, a wide range of conductive materials can be used in electrochemical transducers such as carbon nanotubes (CNTs), gold (Au), zinc (ZnO) and graphene [111].

CNTs, as 1-D nanomaterial, have recently gained a lot of attention as a valuable material for developing wearable electrochemical biosensors [112]. Electrodes for biosensors composed of CNTs are extremely used, since they have advantageous characteristics such as the ability to facilitate redox reactions of various substances, increased dynamic range, sensitivity and detection limit, in addition to promoting thermal, mechanical and chemical stability [113].

Au has been increasingly employed for electrochemical biosensor mechanisms due to the its stability in body fluids and the ability to immobilize target analytes on its surface of in order to quantify their concentrations [23,111,112].

ZnO presents characteristics that make it an excellent candidate material for applications of textile biosensors. It exhibits a high sensitivity to adsorbed molecules, an easy formation of nanostructures, rapid electron transfer and excellent preservation of the stability of biomolecules linked [23]. However, it is important to consider that ZnO nanoparticles have shown some toxic effects on living cells [114].

Graphene is a two-dimensional allotrope of carbon. Graphene-based materials can be moderately hydrophilic (e.g., graphene oxide) or hydrophobic (e.g., reduced graphene oxide). Those with hydrophobic properties can accumulate on cell membrane surfaces causing toxic effects that are much higher in comparison to most of the hydrophilic forms [115]. This material has emerged as an important tool for the study and application of biosensors. It has extraordinary electron transfer capabilities, excellent electrical conductivity, large specific surface area and good biocompatibility [116]. Because of its superior mechanical properties and high flexibility, making it more appropriate for use in wearable electrochemical biosensors. High electrode surface area of graphene can be used to detect small biomolecules such as DNA, gaseous elements and heavy metal ions. Compared with CNTs, limited research has been carried out on graphene/conducting polymer nanocomposites and graphene/carbon paste electrode so there is still scope for further research.

The most commonly used conductive inks for the screen-printing process are silver and carbon inks. Silver ink is mostly printed as a conductive strip (electrode of reference and electrical contacts), while the working (transducer) and auxiliary electrodes are printed mainly through carbon, graphite, graphene, zinc or gold inks and nanomaterials. These materials enable a greater efficiency in immobilizing the bioreceptor and accelerate the transfer rate of electrons on the electrode surface. Consideration should be given to some unknown substances in the inks that can induce unpredictable influences on detection and analysis [106].

The conductivities of electrodes printed on textile substrates depend on factors such as energy surface, porosity and degree of tightness. The substrate must have high chemical stability, compatibility with different paints and additives, resistance to electrolytic media, as well as resistance mechanics. Substrates such as PES; PVC, PP and ceramics have been widely used in the literature [117].

**Figure 10:**
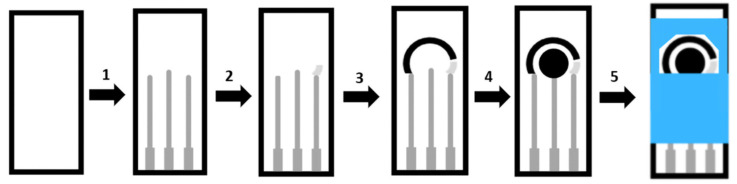
Screen Printing process to produce a biosensor. 1—printing of electrical contacts; 2—printing of RE; 3—printing of AE; 4—printing of WE; 5—printing of insulating layer [118].

The screen printing involves different steps, as shown in Figure 10.

As shown in step 1, we start by printing the first layer of conductive ink on the substrate in order to check the electrical contact between the successive inks of the electrodes. Ag ink has a low cost, presents a good electrical conductivity, being the most used for the construction of electrical contacts of the SPE [119]. To print the following layer, in 2, it is necessary to place a screen with a new defined area on the substrate already with the layer of contacts. This layer will define the reference electrode, the Ag/AgCl conductive ink being the most commonly used. As demonstrated in 3, the ink of interest is printed on a new screen to form the auxiliary electrode. Then, in 4, the same procedure is performed to print the working electrode. It is necessary to consider that after each ink printing to form either the electrical contacts or the different electrodes, it is necessary to solidify the ink through the process of drying (curing). The final stage, in 5, concerns the printing of a layer of insulating ink in order to define the electrical contact area, as well as the surface area of the electrodes, at the other end [120].

The regions that perform active functions correspond to those without the insulating layer, such as the area containing the three electrodes that will be in contact with the sample to be analyzed and the final region of the electrical contacts that will be connected to the potentiostat.

It is necessary to consider some factors that can influence the printing process, such as the paint composition, viscosity, heat treatment and spreading force. The adhesion of conductive inks to the substrate is a determining factor for success in terms of experimental results. Poor adhesion will result in paint delamination, increased strength and, consequently, a decrease in the conductive properties. Thus, it is necessary to think about different solutions in order to obtain a good adhesion between the ink and the substrate. The functionalization of surface allows to confer new properties to the textile material, being the plasma treatment one of the most used methods [121]. During plasma treatment polar groups can be created which increase the surface energy of the substrate and thus improve adhesion to its surface [122,123,124]. It is an environmentally friendly method with high potential for modifying surface of different materials without altering their intrinsic properties [125]

In order to improve the electrochemical properties of the sensor, ensure adhesion and dispersion efficiency of the material on the substrate, some additives can be added to the paints, such as binders and plasticizers. The most used binders are epoxy resins, polyurethane resins, phenolic compounds or cellulose acetate in order to homogenize the constituents of paints. Plasticizers have as main function to provide adhesion of the ink to the substrate, the most common being the 2-nitrophenyl octic ether (o-NPOE); dibutyl phthalate (DPB); dioctyl sebacate (DOS) and tricresyl phosphate (TCP) [123]. Most additives require specific curing temperature conditions for the sensor is not destroyed or show negative effects. Thus, the choice of additives has to be performed as a function of the appropriate temperature of the ink drying [126].

In order to keep all ink components in their liquid form and with adequate viscosity solvents such as acetone and cyclohexanone can be mixed. The choice of this component depends on the application and the print surface. The use of cellulose acetate prevents the formation of cracks if the electrode is flexed [127].

The spreading force of the paint must be carried out with the minimum values, since an excess of speed or pressure can cause failures in the stencil’s filling capacity, influencing negatively the predetermined thickness [128].

It is important to evaluate the surface morphology and topography of screen-printed electrodes before and after immobilization of the bioreceptor. Techniques such as SEM (scanning electron microscope) and AFM (atomic force microscopy) provide both qualitative and quantitative information on many physical properties including size, morphology, surface texture and roughness [129].

## 10. Outlook and Future Scope

The textile sector is a traditional industry and its growth depends on the industry’s ability to innovate. It is important to reinvent this industry, in order to maximize its potential and remain competitive in the market. The key is to foster innovation to add value, always responding to the consumer’s needs and trends. The demand for the creation of smart materials and intelligent textiles grows exponentially all over the world. Hence, this review investigated the state-of-the-art in sweat textile based biosensors, specifically to detect alcohol, and intends to motivate the reader to research, presenting several hypotheses of innovation with the aim of enriching the textile industry by developing new textile-based biosensors with the aim of improving the quality of human life, namely alcohol biosensors to reduce Drunk Driving events, road accidents and subsequent premature death or disability of drivers, passengers and pedestrians.

The development of biosensors as non-invasive devices for easy monitoring and rapid assessment of an individual’s health status has been one of the great ventures in the scientific community over the past years. In this review article, we have discussed research projects related to biosensors capable of detecting ethanol in sweat, as well as detecting viruses and diseases like diabetes, heart/circulatory failure, metabolic/respiratory disorders among others. However, this is an immature market niche, so it’s necessary take a chance on new methodologies and try to create innovative and functional products that attract the interest of the public.

Yet, the long-term durability and reproducibility of biosensors require further research efforts. The durability of a biosensor is often compromised, mainly by the type of bioreceptor used, that is, by the element that provides selectivity to the biosensor. Usually, the integration of bioreceptors such as enzymes, antibodies and aptamers into biosensors causes low stability and durability, since they end up having little resistance to adverse environments such as high temperatures, high pressure, and pH variations. Thus, in order to solve this problem, an alternative technique called Molecular Printing has recently emerged, allowing the creation of these structures in an artificial way. This technology, compared to the bioreceptors mentioned above, has advantages such as low cost, robustness, high stability, long-term durability (months/years), and selectivity. The wide range of scientific expertise involved in MIP development bodes well for the future of the science. While more and more MIP-based biosensors are proposed, featured with significant accuracy, they can be feasibly applied for various applications such as medical diagnosis, drug delivery, environmental monitoring by gas sensing, food safety, among others. So, it is important to explore this technique in order to make a substantial advance in the performance of (bio)sensors. Furthermore, the transduction mechanism needs to be improved by using novel materials. The use of carbon materials or metal nanoparticles will contribute to a better electron transfer which, in turn, will lead to an increase in the sensitivity of the device.

Despite all the non-invasive advantages, the diagnosis of human sweat on the skin surface of an individual may have some challenges such as low sample volumes, contamination of the skin and the possibility of evaporation. These factors should be strictly studied in order to produce accurate and efficient alcohol devices, which could be used in automotive as security systems and in medical field to control abstinence/alcohol diseases, etc.

Further, the discussed sweat-based wearable electrochemical biosensors require seamless integration with smartphone apps for continuous tracking and display of the BAC, along with the appropriate data security and privacy. The future of wearable alcohol sensing devices is bright, since it shows promising capabilities relying on successful performance validation in large-scale on-body trials relative to concurrent changes in BAC as well as the integration of such devices into textile-based platforms for point-of care testing.

Overall, there are a lot of potential in healthcare flexible biosensors, with major advances being made in the non-invasive monitoring of new biomarkers, which can range from electrolytes/metabolites to bacteria and proteins/hormones. The increased accuracy, selectivity and utility of wearable biosensing platforms are enhancing their commercial impact. Hence, to ensure high efficiency, additional efforts should be made to improve the validation of sweat biosensors. It is important to consider factors such as the design, microfluidic sampling and transport systems, along with system integration and miniaturization combined with flexible materials for enhanced wearability and ease of operation.

Overall, in view of sweat biosensors potential, besides ethanol detection to monitoring alcohol consumption, it is expected that these devices will find increasing uses in medical field applications, which help doctors and patients for various purposes such as control of illness, clinical care, preventive treatment, patient health information, and disease reviews. A focus on qualities as long-term durability, specificity, sensitivity, affordability, simplicity, and portability will increase the probability of these innovative products finding their place in the real world.

## Figures and Tables

**Table 1:** Overview of wearable/flexible biosensors to detect alcohol in sweat.

Device	Localization	Testing Status and Availability	Ref.
SCRAM^TM^ produced by Alcohol Monitoring Systems (AMS) in Littleton, USA.	Ankle	It is the most widely used wearable device in clinical research trials and has been adopted internationally by justice systems for court monitored sobriety since 1997, being the most representative alcohol biosensor nowadays.	[54]
WristTas^TM^ produced by GINER lab, in Newton, MA	Wrist	It was developed primarily for use in medical settings with more compliant subjects, but lacks the protocols for detecting results‘ tampering and has not yet been adapted for court use. Completed laboratory testing has been done to the device, but it is not currently commercially available.	[55]
BACtrack Skyn produced by BACKtrack in San Francisco, California	Wrist	Newest generation of wrist biosensors. Is a bracelet capable of measuring alcohol levels through insensible sweat. Commercially available since 2015.	[56]
Proof^TM^ produced by Milo Sensors in Santa Bárbara, California, USA	Wrist	Bracelet that utilizes an enzymatic electrochemical biosensor cartridge for alcohol detection, which can be coupled to a Smartphone App that targets safe recreational alcohol consumption with an integrated social aspect. Discontinued after laboratory testing.	[57]
Quantac Tally produced by Quantac Inc. in New York, USA	Wrist	Combines alcohol monitoring data in its coupled smartphone App with health-related metrics to inform the wearer of personalized insights into health impacts of their alcohol consumption. Discontinued after laboratory testing.	[58]
Iontophoretic Biosensing System produced by Kim et.al in Departments of Nanoengineering and Electrical & Computer Engineering, University of San Diego, California, USA	Tatto on arm	Screen-printed commercial tatto paper with silver and silver chloride electrodes. The electronic system transfer the results via Bluetooth via the wearer’s mobile device. Currently in laboratory testing.	[59]
AlcoWear produced by McAfee et al. in San Francisco, California, USA	Wrist	Gait smartphone AlcoGait application paired with any smart watch to measure accelerometer and gyroscope. Currently in laboratory testing.	[60]
Sensor-equipped Smart Shoes produced by Eunjeong Park and his team, in University Departments of Los Angeles, California/Boston, Massachusetts, USA and Seol, Korea	Shoes	Gait using pressure sensors inserted in insole of shoe. Currently in laboratory testing.	[61]

**Table 2:** Flexible biosensors to detect alcohol in sweat.

Platform	Target Analyte/Bioreceptor	Measurement Technique	Linear Range	Ref.
Electrochemical biosensor: flexible co-planar Au or ZnO integrated in PI from bonding	EtG/EtG antibody	Electrochemical Impedance Spectroscopy (EIS)	2 × 10^−6^–2.17 mM	[23]
Optical biosensor: polyethylene strip composed of two enzymes	Ethanol/Alcohol Oxidase (AOx) and Horseradish peroxidase (HRP)	Chronoamperometry	0–54.23 mM	[48]
Electrochemical biosensor: hydrogel adhesive with screen printed electrodes	Ethanol/AOx	Chronoamperometry	3.0–36.0 mM	[59]

**Table 3:** Advantages and disadvantages of the electrochemical techniques.

Method	Description	Advantages	Disadvantages	Ref.
Potentiometry	It is based on the measurement of the potential difference of the working electrode and the reference electrode. This potential difference is formed when, for example, an antigen-antibody interaction occurs, and it is measured under practically zero current conditions.	- Simple dectection scheme and signal processing; - Suitable for detecting analytes in the Mm concentration range.	- Requires more time and cost of analysis.	[64,67,69]
Chronoamperometry	Direct measurement of the redox reaction current under a constant potential applied to the working electrode. The measured current is the rate of transferred electrons as a function of time, being proportional to the concentration of the target analyte.	- Simple detection; - Easy post-processing to convert analyte concentration into electrical current; - Mediators can be used in order to reduce the necessary potential and, therefore, energy consumption.	- -Usually an enzyme is needed to provide selectivity; - -It may result in an inaccurate concentration conversion if the linear range of the analyte to be detected is below µM.	[43,69,74]
Voltametry	It consists of applying a potential in the electrochemical cell, measuring the resulting current. Voltammetric methods can be divided into Cyclic Volametry (CV); Differential Pulse Voltammetry (DPV); Linear Scanning Voltammetry (LSV); Square Wave Voltammetry (SWV).	- Ability to extract multiple analytes at once; - It obtains specific qualitative and quantitative information about the species involved in the redox reaction; - Good ability to detect drugs, hormones and heavy metals.	- Complex post-processing when compared with chronoamperometry to extract and identify peaks corresponding to the desired analyte;	[69,74,75,76]
Elechtrochemical Impedance Spectoscopy	Electrode impedance measurement. It characterizes the structure and function of electrodes, especially those that have been modified with biological material.	- Rapid technique for characterizing the structure and functional operation of electrodes using biomaterials; - Good ability to detect drugs, hormones and heavy metals	- Long analysis times; - Post-processing more complex than voltammetry; - It may have low sensitivity, being necessary to include amplification techniques.	[69,75,77]
Conductimetry	It measures the variation in electrical conductivity that occurs in biological processes and that is caused by changes that occur in the concentration of ionic species in solution.	- Possibility of monitoring changes in electrode conductance.	- Mostly used with enzymes; - Difficulty in performing.	[43,69]

**Table 4:** Immobilization techniques.

Technique	Caracteristics	Advantages	Disadvantages	References
Adsorption	- Weak forces such as Van der Wall Forces; Hydrogen Bonding; Hydrophobic or Electrostatic Interactions	- Simplicity - Low cost - Wide range of support materials	- Harsh environments could lead to desorption from the bioreceptor - It does not be able to control the orientation of the bioreceptor on the surface	[92,93]
Crosslinking	- Bond between bioreceptor/cross linker	- -Simplicity - -Irreversible binding - -High surface coverage	- Loss of activity due to structural rearrangements; - Toxicity	[91,93,94]
Entrapment	- Incorporation of the bioreceptor within a gel or polymer	- Stability and protection of the bioactive agent against degradation	- Diffusional limitations; - Possibility of biomolecule leakage	[93,95,96]
Covalent	- Covalent bonds are in general formed between side-chain-exposed functional groups of modified supports	- Strong binding - High stability - Most feasible for long term use	- Irreversible binding; - Complexity and cost. Risk of activity loss during immobilization.	[93,97,98]

**Table 5:** Represents the list of various materials employed in the development of flexible biosensors. Note_ N/I means No Information.

Biosensor	Target Analyte	Bioreceptor	Materials	Linear Range	LOD	Ref.
Monitoring alcohol consumption through sweating	EtG	Antibody EtG	- Coplanar electrodes of gold and zinc oxide; - Polyamide (PA) substrates; - Glass substrates;	0.001–100 μg/L	(Au) 1 μL/L (ZnO) 0.001 ug/L	[23]
Applications in detecting the point of exposure to Influenza A virus	Influenza A	Antibody specific for the H1N1 Influenza A protein	- Conductive electrodes of silver (Ag); - Graphene oxide (GO) transduction film; - PA and Cotton (CO) substrates.	10 ng/mL– 10 μg/mL	10 ng/mL	[103]
Detection of redox active biomolecules in biological fluid with a textile organic electrochemical transducer	Adrenaline; Dopamine; Ascorbic Acid	N/I	- Electrodes of PEDOT:PSS; - CO and - Lycra substrates.	Adrenaline: 10^−4^–10^−2^ (M) Dopamine: 3.10^−5^–5.10^−4^ (M) Ascorbic Acid: 2.10^−6^–3.10^−5^ (M)	Adrenaline: 0.78 ± 0.05 × 10^−8^ (M) Dopamine: 0.8 ± 0.1 × 10^−8^ (M) Ascorbic Acid: 1.1 ± 0.1 × 10^−8^ (M)	[104]
pH monitoring to detect diseases such as diabetes	Glucose	Glucose Oxidase Enzyme	- RE of - (Ag/Ag/Cl) - WE of graphite; - Substrate of 55% Cellulose/45% PES	N/I	N/I	[105]
Monitoring the lactate level in sweat to detect diseases such as heart/circulatory failure, metabolic/respiratory disorders	Lactate	Lactate Oxidase Enzyme	- AE of Au elastomeric fiber; - Au/Ag/AgCl RE with PVB (polyvinyl buturial) couting; - WE of gold fiber followed by a layer of chitosan (CS)	0 mM– 30 mM	0.137 mM	[106]
